# Retraction: CircRNA PVT1 modulates cell metastasis *via* the miR-181a-5p/NEK7 axis and cisplatin chemoresistance through miR-181a-5p-mediated autophagy in non-small cell lung cancer

**DOI:** 10.1039/d1ra90075g

**Published:** 2021-02-03

**Authors:** Laura Fisher

**Affiliations:** Royal Society of Chemistry Thomas Graham House, Science Park, Milton Road Cambridge CB4 0WF UK advances-rsc@rsc.org

## Abstract

Retraction of ‘CircRNA PVT1 modulates cell metastasis *via* the miR-181a-5p/NEK7 axis and cisplatin chemoresistance through miR-181a-5p-mediated autophagy in non-small cell lung cancer’ by Limin Yan *et al.*, *RSC Adv.*, 2019, **9**, 42324–42334, DOI: 10.1039/C9RA08872E.

The Royal Society of Chemistry hereby wholly retracts this *RSC Advances* article due to concerns with the reliability of the data. The images in the article, and the raw data provided by the authors, were screened by an image integrity specialist. The western blots in the paper all look very similar to each other, the bands have a regular shape throughout and do not appear genuine. In addition, the raw data does not look genuine, it is cropped and lacks molecular weight markers. Therefore, the data provided by the authors cannot be accepted as raw data and does not validate the published data in the article.

Furthermore, the blots and many other features of the article were found to very closely resemble blots and features from a number of other articles, which is unexpected given that there are completely different author lists for these articles.

Given the significance of the concerns about the validity of both the data in the article and the raw data provided by the authors, the findings presented in this paper are not reliable.

Zhiyong Zhang does not agree with the retraction. The other authors were informed but have not responded to any correspondence regarding the retraction.

Signed: Laura Fisher, Executive Editor, *RSC Advances*

Date: 19^th^ January 2021

## Supplementary Material

